# Earth’s volatile depletion trend is consistent with a high-energy Moon-forming impact

**DOI:** 10.1038/s43247-023-00694-9

**Published:** 2023-02-16

**Authors:** Natalia V. Solomatova, Razvan Caracas

**Affiliations:** 1 CNRS, Ecole Normale Supérieure de Lyon, Laboratoire de Géologie de Lyon LGLTPE UMR5276, Centre Blaise Pascal, 46 allée d’Italie, Lyon, 69364 France; 2 Université de Paris, Institut de Physique du Globe de Paris, CNRS, 1, rue Jussieu, Paris, 75005 France; 3 The Center for Planetary Habitability (PHAB), University of Oslo, Blindern, Oslo, Norway

**Keywords:** Geochemistry, Geochemistry, Inner planets, Early solar system

## Abstract

The abundance of volatile elements in the silicate Earth relative to primitive chondrites provides an important constraint on the thermochemical evolution of the planet. However, an overabundance of indium relative to elements with similar nebular condensation temperatures is a source of debate. Here we use ab initio molecular dynamics simulations to explore the vaporization behavior of indium from pyrolite melt at conditions of the early magma ocean just after the Moon-forming impact. We then compare this to the vaporization behavior of other minor elements. When considering the volatility of the elements from the magma ocean in the absence of the solar nebula gas, we find that there is no overabundance of indium. On the contrary, there is a slight deficit in the abundance of indium, which is consistent with its moderately siderophile nature. Thus, we propose that a high-energy Moon-forming impact may have had a more significant contribution to volatile depletion than previously believed.

## Abstract (web summary)

Volatile loss during the Moon-forming impact can explain the observed depletion trend in Earth’s mantle, according to atomistic simulations of magma ocean degassing.

## Introduction

During planetary accretion, physical and chemical processes, such as melting and vaporization, result in the loss and fractionation of elements. The degree to which elements are lost from planetary bodies generally correlates to their level of volatility. The volatility of an element describes its predisposition to exist in the gaseous state relative to a liquid or solid and is often defined by the temperature at which 50% of the element is condensed in the solar nebula gas at 10^−4^ bar (“the 50% condensation temperature”). The condensation temperature is inversely related to the element’s level of volatility and is often considered in the context of elemental abundances in the bulk silicate Earth (BSE). The abundance of elements in the BSE relative to primitive CI chondrites gives information about post-nebular thermochemical events in Earth’s history that have altered the elemental proportions. While refractory lithophile elements, such as Ca and Al, exist in the BSE in approximately chondritic quantities relative to Mg, the more volatile lithophile elements progressively decrease in abundance in the BSE relative to CI chondrites. Siderophile elements generally fall below the volatile depletion trend due to their assumed sequestration into the core^1^.

Some volatile elements appear to fall above the volatility trend; the reason for their apparent overabundance has been a topic of debate^2–8^. In recent years, indium has been under particular scrutiny due to its overabundance in the BSE relative to elements with similar condensation temperatures while often possessing a more siderophile nature^1,2,9,10^. Several explanations, which are not necessarily mutually exclusive, have been proposed:

*Hypothesis 1. Indium may have been less volatile in the inner solar nebula where Earth accreted*^2^. Analysis of the volatile depletion trend comes with the assumption that the 50% condensation temperatures from a predefined solar nebula gas represent the vaporization behavior of elements during Earth’s accretion. However, it is likely that the conditions of the solar nebula were not homogenous. Based on a reassessment of the relative abundance of In in mantle rocks and metal–silicate partitioning data, Wang et al.^2^ propose that In may have been more refractory than Cd and Zn during the formation of the Earth. The authors suggest that the volatility of elements was not constant throughout the solar nebula and that the Earth was accreted from materials that do not represent existing chondrites today.

*Hypothesis 2. Similarly volatile elements were more siderophile than In at early Earth conditions and so were more significantly sequestered into the core*^3^. It is possible that In behaved in a much more lithophile way compared to Cd, Sn, and Pb during Earth’s accretion, resulting in more significant sequestration of Cd, Sn, and Pb into the core compared to In. In this scenario, a linear trend can then be drawn through In and the halogens, also conveniently removing the apparent overabundance of halogens in the SE.

*Hypothesis 3. Previous and/or current estimates of the condensation temperature of In are incorrect*^4^. It is possible that the 50% condensation temperatures in the solar nebula gas and abundances in the BSE, particularly of the trace elements, are not very accurate. For example, improved estimates of the condensation temperatures that consider a non-ideal mixing nature of trace elements in Fe-rich metals and sulfides place In along the volatility trend along with the halogens^4^; however, the modified volatility trend leaves behind some of the lithophile elements, such as Zn, Cs, and Rb. It is possible that further refinements of the 50% condensation temperatures and/or abundances would result in a smoother process-driven volatility trend.

*Hypothesis 4. The volatility trend should not be a continuous linear trend in the first place*^5,6^. It has been argued that we should not expect the abundance of elements in the BSE to continuously and linearly decrease with increasing volatility. Instead, the volatility trend should follow a hockey-stick pattern where the volatility of elements plateaus above a certain condensation temperature due to the addition of 10–15 wt% CI-chondrite material. The hockey-stick volatile depletion pattern is well-documented in CV, CM, and CR chondrites^5,11,12^ and may have imprinted the behavior in the Earth’s volatile abundances.

*Hypothesis 5. The volatile depletion trend was more strongly affected by the melting and vaporization processes after the Moon-forming impact than during accretion*^7^. It has thus far been assumed that the accretion of Earth was the main process that imprinted Earth’s volatile depletion trend; however, the Moon-forming impact, which likely melted and vaporized the entire planet, had a significant contribution to the volatile depletion trend. Vaporization experiments on reduced silicate melts containing a range of minor volatile elements demonstrate that the volatility of In is consistent with the depletion trend when plotting the abundances against the proportion of volatiles remaining in the silicate melt instead of the condensation temperatures from a nebular gas^7^. It is well established that element volatilities from silicate melts are different from those during solar nebula condensation^13–15^. The fact that the abundance of In is consistent with vaporization from a reduced silicate melt in the absence of a nebular gas suggests that the Moon-forming impact may have been responsible for the abundance of In and other moderately volatile elements in the BSE.

It is possible that, to varying extents, all five explanations are responsible for the apparent overabundance of In. Understanding the influence of each process is essential for quantifying the volatile distribution on Earth. Here, we explore the latter hypothesis by calculating the vaporization behavior of volatile elements from a pyrolite melt that represents the BSE composition^16^ in the absence of the solar nebula gas (see Supplementary Tables 1 and 2 for compositional details of all the melts examined in this study). It is important to note that although In likely existed in the +2 oxidation state during Earth’s accretion in the presence of the highly reducing solar nebula gas^4^, In probably existed in the +3 oxidation state in the magma ocean after the Moon-forming impact (see Supplementary Note 1). Additionally, the presence of S may have affected the vaporization behavior of In due to its chalcophile nature. Thus, we examine the volatility of In with respect to its oxidation state and in the presence and absence of S. The concentrations of the trace elements are greatly exaggerated with respect to nominal concentrations for computational feasibility and to achieve statistically meaningful results (see Supplementary Note 2). The effect of concentration on the elemental vaporization rate from pyrolite melts has been previously explored^17^.

## Results

### Volatility changes with density

**Fig. 1:**
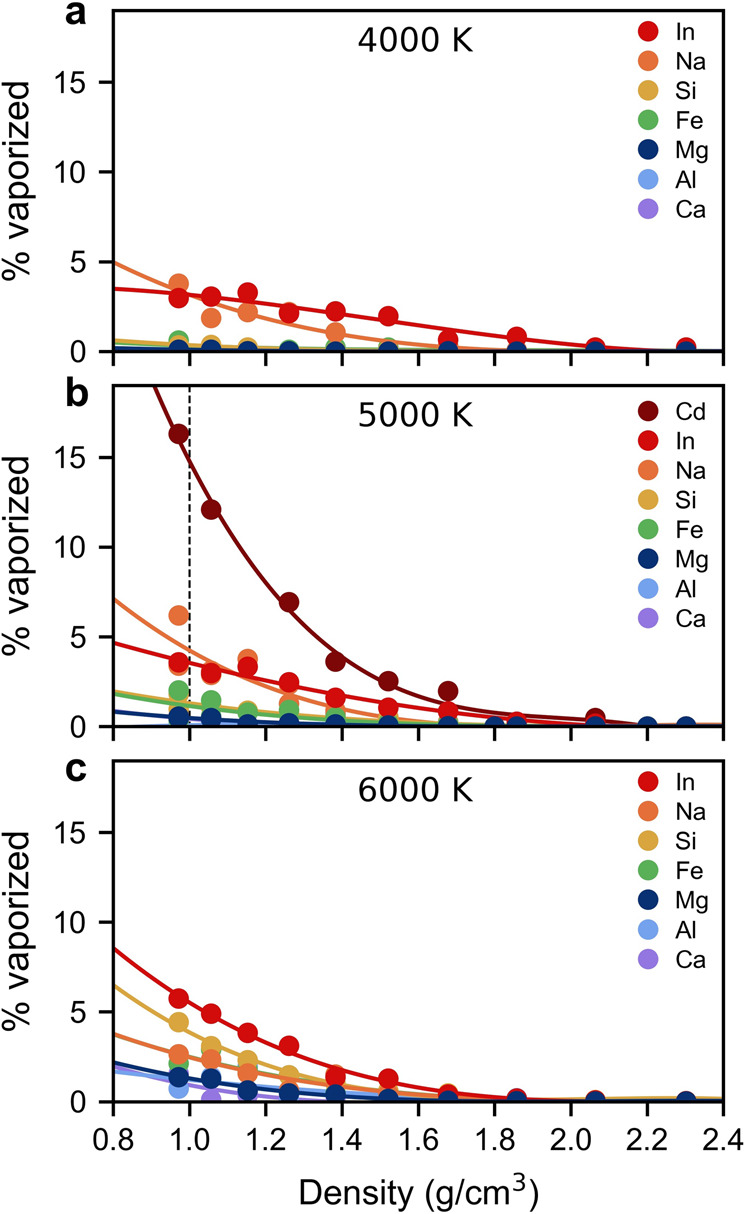
Volatility as a function of density and temperature. The proportion of elements existing in the vapor phase in In^3+^-bearing pyrolite at **a** 4000 K, **b** 5000 K, and **c** 6000 K. The calculated volatilities for Cd^2+^-bearing pyrolite are shown at 5000 K as well. A single polynomial was fitted to each element type (averaged for the two compositions at 5000 K) as guide for the eyes to show the increasing vaporization trend with decreasing density. A dashed vertical line at 5000 K shows the conditions explored in Fig. 3.

First, we calculate the proportion of elements vaporized from In^3+^-bearing pyrolite melt into the gaseous phase as a function of density and temperature. At a density of about 2.6 g cm^−3^ nano-sized cavities begin to spontaneously nucleate into which volatile species are free to vaporize^17^. The predisposition of an element to exist in the vapor phase with respect to the melt phase depends on the density, temperature, and the element’s level of volatility. In the In^3+^-bearing pyrolite system, volatile species begin to vaporize into the nanocavities at ~1.8 g cm^−3^ (Fig. 1) and the fraction of volatile elements in the vapor phase relative to the melt phase increases with decreasing density (i.e., decreasing pressure).

At 4000 K, Si, Fe, Mg, Al, and Ca remain almost entirely in the silicate melt up to the lowest density explored and only Na and In are vaporized into the nanocavities. At 5000 K, Mg, Fe, and Si begin to vaporize in measurable quantities (1–2% exist in the vapor phase) while Al and Ca reside in the melt. It is not until the temperature increases to 6000 K that Al and Ca begin to vaporize from the melt in proportions larger than 1%. At the conditions explored in this study, the volatility of In is greater than the volatilities of Si, Fe, Mg, Al, and Ca. At temperatures of 4000–5000 K, the volatility of In is similar to Na (e.g., 3–4% of In and Na exist in the vapor phase at 1 g cm^−3^) while at 6000 K, the volatility of In is systematically higher than that of Na (e.g., 6% of In and 2.5% of Na exist in the vapor phase at 1 g cm^−3^). At 6000 K, the volatility of Si surpasses the volatility of Na, reaching about 4% by 1 g cm^−3^. Thus, temperature and density have strong effects on volatility behavior, suggesting that the composition of the early atmosphere after the Moon-forming impact would have evolved rapidly over time.

### Volatile element depletion trend

**Fig. 2:**
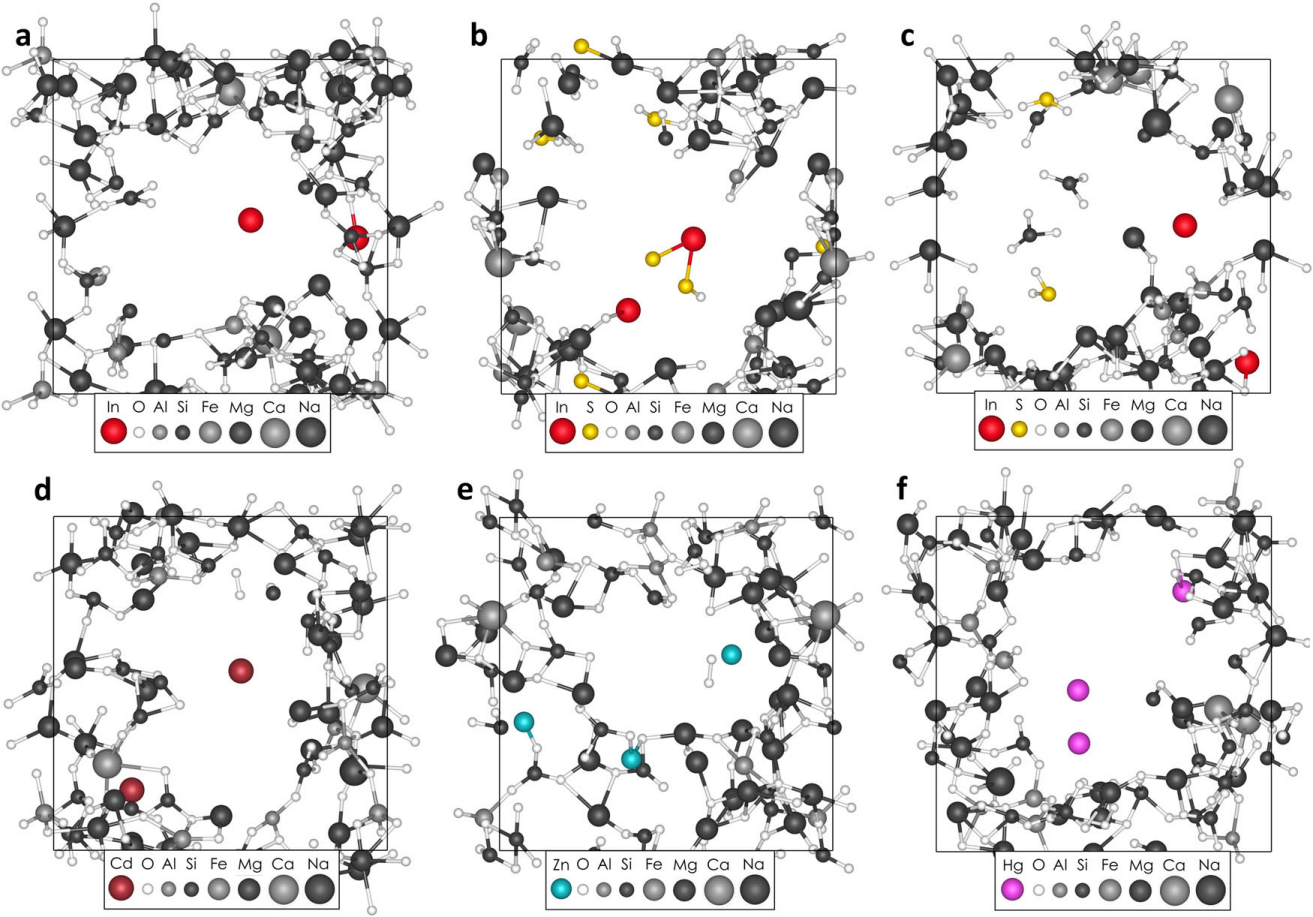
Snapshots of the vaporization of In, Cd, Zn, and Hg from pyrolite melts at 5000 K and 1 g cm^−3^. **a** Vaporization of In^3+^ (red) in the absence of S. **b** Vaporization of In^3+^ (red) in the presence of S^2−^ (yellow). **c** Vaporization of In^3+^ (red) in the presence of S^6+^ (yellow). The oxidation states of In and S are described as they were initially added to the silicate melts (e.g., In_2_O_3_) and are not constrained during the simulation. **d** Cd-bearing pyrolite melt with Cd, O_2_, and SiO species in the vapor phase. **e** Zn-bearing pyrolite melt with Zn and O_2_ species in the vapor phase. **f** Hg-bearing pyrolite melt with two Hg atoms in the vapor phase.

We further compute the vaporization behavior of In added to pyrolite melt in the +1, +2, and +3 oxidation states, in the presence and absence of S^2−^, at 1 g cm^−3^ and 5000 K. We also calculate the vaporization behavior of In^3+^ in the presence of S^6+^. See Fig. 2a–c for snapshots of the vaporization behavior of In^3+^ in the absence and presence of S^2−^ and S^6+^. To constrain the volatile depletion trend, we compare our results on In with additional calculations on pyrolite melts containing Zn^2+^, Cd^2+^ and Hg^2+^ at the same density–temperature conditions. We evaluate the volatility trend with respect to the 50% condensation temperatures in the solar nebula gas and the elemental abundances in the bulk silicate Earth (BSE) relative to CI chondrites.

We choose Zn and Cd for their similar 50% condensation temperatures to In, and we choose Hg to extend the volatility space to higher volatilities. See Fig. 2d–f for visualizations of their vaporization behavior. We calculate the volatility of Cd^2+^ from pyrolite melt and compare its volatility to In^3+^ as a function of density at 5000 K. We find that Cd^2+^ is much more volatile than In^3+^ (e.g., 15% of Cd^2+^ and 4% of In^3+^ are in the vapor phase at 1 g cm^−3^) at all densities explored in this study. Furthermore, the volatility of Cd^2+^ increases rapidly with decreasing density, at a much steeper rate than In^3+^. Thus, the Cd/In ratio in the vapor phase will increase with decreasing pressure and decreasing thickness of the atmosphere.

**Fig. 3:**
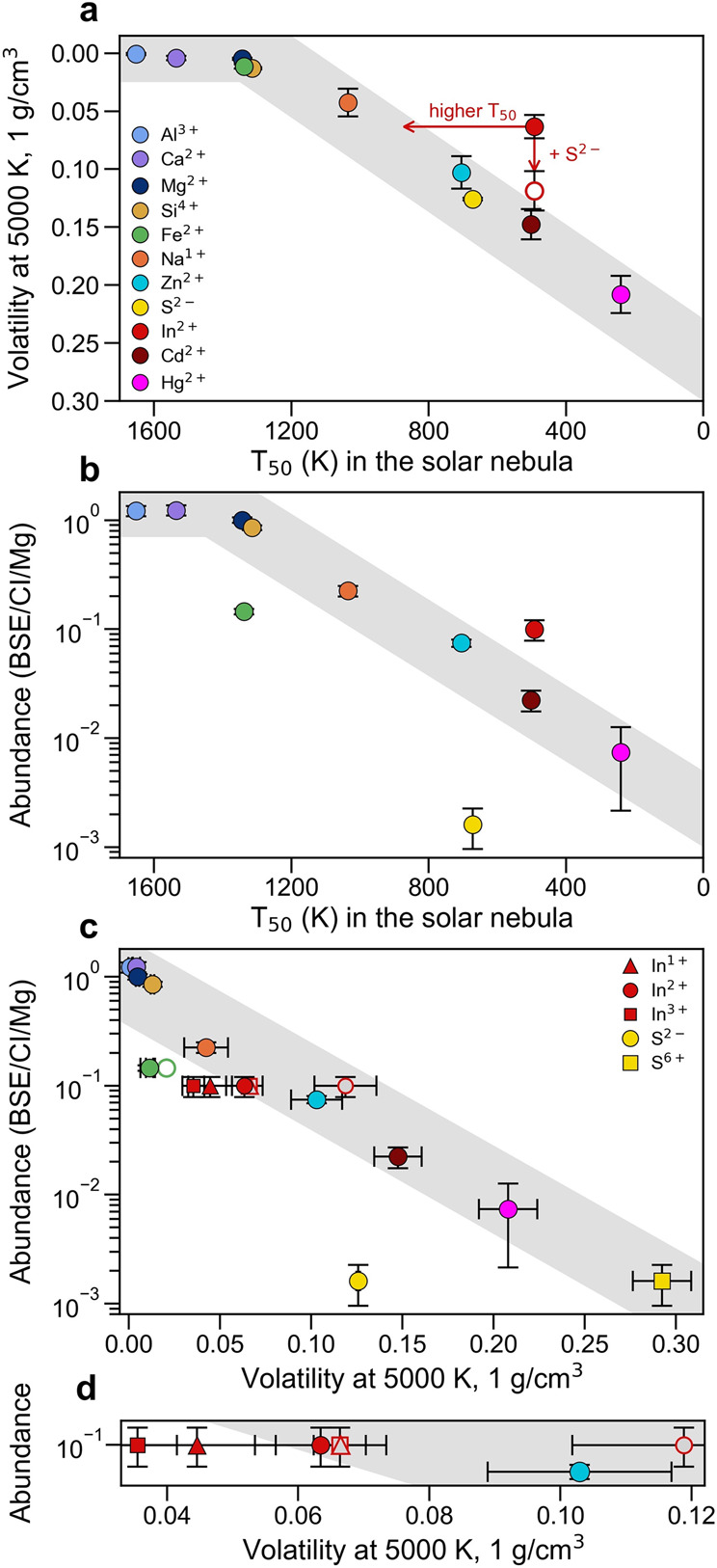
Volatile element depletion trends. **a** The volatility of elements from pyrolite melt calculated from the ab initio molecular dynamics results of this study at 5000 K and 1 g cm^−3^ against the 50% condensation temperatures in the solar nebula gas^4^, demonstrating the linear relationship between the volatility of the elements from pyrolite and the 50% condensation temperatures in the solar nebula gas. Here, volatility is defined as the proportion of the volatile elements existing in the vapor phase in equilibrium with pyrolite melt. **b** The abundance of the elements in the bulk silicate Earth (BSE) relative to CI chondrites, normalized to the abundance of Mg^19^, as a function of the 50% condensation temperatures in the solar nebula gas^4^. **c** The abundance of the elements in the BSE as a function of their volatility as calculated from the ab initio molecular dynamics simulations of this study at 5000 K and 1 g cm^−3^. The volatilities of In^1+^ (triangle), In^2+^ (circle), and In^3+^ (square) are shown in the absence of S^2−^ (solid red symbols) and in the presence of S^2−^ (open red symbols). **d** Magnification of the region with In^1+^, In^2+^, In^3+^, and Zn^2+^ of part (**c**). Gray bands are guides for the eyes. Uncertainties on computed volatilities correspond to the convergence window of the volatilities with simulation time.

Figure 3a demonstrates the correlation between two ways of quantifying “volatility” (1) the 50% condensation temperature in the solar nebula gas^4^ and (2) the proportion of volatile elements in the vapor in the melt-vapor pyrolite system at 5000 K and 1 g cm^−3^ (this study). As would be expected, the volatility of the elements (the proportion existing in the vapor phase) and their 50% condensation temperatures in the solar nebula gas are negatively correlated: higher volatilities are associated with lower condensation temperatures (Fig. 3a). The volatility of elements with 50% condensation temperatures above that of magnesium are expected to remain in the melt and have a volatility of 0%, as shown by the plateau between 1300 and 1650 K in Fig. 3a. Below temperatures of 1300 K, there is an approximately linear increase in the degree of volatility with decreasing 50% condensation temperatures in the solar nebula gas. In Fig. 3, we use the 50% condensation temperatures from the reappraised temperatures of Wood et al.^4^; see Supplementary Fig. 1 for the volatility trends with respect to the 50% condensation temperatures of Lodders^18^.

While the volatilities of Hg^2+^, Cd^2+^, and Zn^2+^ that we obtain from our simulations fall along the linear volatility–*T*_50_ trend, we find that the volatility of In^2+^ is lower than would be expected based on its 50% condensation temperature in the solar nebula gas. In other words, the volatility of In^2+^ does not fall along the linear volatility–*T*_50_ trend of Fig. 3a (see Supplementary Table 3 for the 50% condensation temperatures in the solar nebula gas and Supplementary Table 4 for the volatilities calculated in this study). Due to the very similar calculated 50% condensation temperatures for In^2+^ (492 K) and Cd^2+^ (502 K) dissolving into FeS^4^, one may expect that their vaporization behavior from pyrolite might be similar as well; however, the volatility of Cd^2+^ (~15%) is more than twice as large as the volatility of In^2+^ (~6%). Thus, it is possible that the relative volatility of In^2+^ in pyrolite melt is simply lower than in the FeS system or that the existing thermodynamic estimates of the 50% condensation temperature in the solar nebula gas are too low^4,18^.

As has been done in the past, we plot the elemental abundances in the BSE relative to CI chondrites^19^ with respect to their 50% condensation temperatures in the solar nebula gas^4^ normalized to Mg in Fig. 3b, showing the apparent overabundance of In from the volatility trend. In Fig. 3c, we show the same elemental abundances plotted against the volatilities calculated in this study from pyrolite melt at 5000 K and 1 g cm^−3^ rather than the 50% condensation temperatures in the solar nebula gas. Unlike in Fig. 3b, here it appears that the BSE has an underabundance of In when considering the In^1+^ and In^3+^ without the presence of S^2−^ within the pyrolite melt. In^2+^ with and without the presence of S^2−^ brackets the volatility trend. The presence of S^2−^ in the melts with In^1+^ and In^3+^ shifts their volatilities to that of the S^2−^-free In^2+^-bearing pyrolite melt.

Considering the chalcophile nature of In, one might expect that the presence of S would increase the volatility of In through the formation of S-bearing gas molecules. Indeed, the presence of S^2−^ in the pyrolite system results in an increase in the volatility of In^3+^ (3.5–6.1%), In^2+^ (6.3–11.9%), and In^1+^ (4.5–6.6%) resulting in the formation of S-bearing species, such as InSO and InSO_2_ (see Supplementary Table 5 for speciation statistics). Conversely, the presence of S^6+^ in the In^3+^-bearing melt results in only a negligible increase in the volatility of In^3+^ (3.5–4.3%). In fact, 10%, 14%, and 3% of the vaporized In^3+^, In^2+^, and respectively In^1+^ atoms, form gaseous species with at least one S atom in the S^2−^-bearing melts. Meanwhile, fewer than 2% of the vaporized In^3+^ atoms form gaseous species with at least one S atom in the S^6+^-bearing melts. Thus, we find that the presence of S in the form of S^2−^ increases the volatility of In^2+^ and In^3+^ with a smaller effect on In^1+^ and the presence of S^6+^ has only a negligible effect on the volatility of In^3+^.

As predicted by the volatile depletion pattern in the context of the 50% condensation temperatures in the solar nebula gas (Fig. 3b), S^2−^ is underrepresented in the BSE when plotted against its volatility from pyrolite melt at 5000 K (Fig. 3c). The effect of oxidizing S appears to decrease the degree of S depletion from the BSE. In the endmember case where all S exists as S^6+^, it is vaporized in much larger proportions (~30% S^6+^ compared to ~12% of S^2−^ existing in the vapor phase), falling perfectly along the volatile depletion trend. The oxidation state of S in silicate melts is strongly dependent on the oxygen fugacity^20–22^ and it is known that the oxidation state of S affects its degassing behavior^23,24^. Thus, the oxygen fugacity of the BSE just after the Moon-forming impact will affect the proportion of S^2−^ and S^6+^ species, which will in turn affect the degree of sulfur vaporization from the magma ocean, the amount sequestered into the iron core, and its effect on the volatility of chalcophile volatiles.

## Discussion

In high-angular momentum simulations of the Moon-forming impact, a planetesimal hits an oblate fast-spinning proto-Earth, resulting in a hot vapor-rich toroidal planetary body, termed the synestia^25,26^. The synestia reaches temperatures of up to 7000 K at the center of the synestia with a vapor mass fraction of >80%^27^. Synestias may also form from the impact of two half-Earths^28^, as well as several other intermediate impact scenarios^26^. Volatile loss may have been substantially lower in a cooler canonical Moon-forming impact, which would have resulted in a 20% vaporization of Earth by mass^29^. The results of our ab initio simulations demonstrate that the Earth’s volatile depletion trend is consistent with the melting and vaporization of Earth that occurred after a high-energy impact. We demonstrate that when considering the volatility trend of the elements from the magma ocean in the absence of the solar nebula gas, there is in fact no overabundance of In. Our prediction that In (in all oxidation states) is less volatile than Zn and Cd, which have similar condensation temperatures to In, is consistent with the experimental results on reduced silicate melts at ~1600 K of Norris and Wood^7^ and is consistent with Hypothesis #5 (see the “Introduction” section). The slight deficit in the abundance of In is consistent with its moderately siderophile nature. In the presence of S, In obtains volatility that falls along the volatile element depletion trend without the need for sequestration into the core.

Without excluding the possibility of a canonical impact or other impact regimes, we demonstrate that the volatile depletion trend is consistent with a high-energy high-temperature Moon-forming impact in the absence of the solar nebula gas, granted that an efficient mechanism for volatile loss was responsible for removing heavier elements. It has long been argued that giant impact(s) may have been responsible for volatile depletion on Earth^30–32^, particularly the late loss of heavier elements^33^. If heavier elements become atmophile^34^, they may be lost by hydrodynamic escape^35^. However, the efficiency of volatile loss by hydrodynamic escape is not yet well understood and is very limited for water^29^. Additional studies on possible volatile loss mechanisms under these conditions are needed. In this study, we demonstrate the ability of ab initio simulations to estimate the volatility of minor and major rock-forming elements at conditions of the early magma ocean and we highlight the need for future ab initio studies on silicate melts incorporating additional moderately volatile elements and examining the effect of melt chemistry (e.g., oxidation state and sulfur content) on their vaporization behavior.

### Computational methods

We perform ab initio molecular dynamics simulations to examine the vaporization behavior of indium and major rock-forming elements from pyrolite melt at temperatures of 4000, 5000, and 6000 K. We use the projector-augmented wave (PAW) method^36^ of density functional theory (DFT) in the Vienna Ab initio Simulation Package (VASP)^37^. The exchange-correlation energy is treated with the generalized gradient approximation (GGA) in the Perdew–Burke–Ernzerhof (PBE) form^38^. Kinetic energy cutoffs of 500 and 800 eV were used for the plane-wave expansion of the wavefunctions and augmentation charges, respectively. Calculations were performed with the NVT canonical ensemble, where the number of atoms (*N*) and the supercell volume (*V*) is fixed, and the temperature (*T*) is regulated with a Nosé–Hoover thermostat^39,40^. A time step of 1 fs is used for all calculations and the duration of the simulations is 10–50 ps. The Brillouin zone is sampled at the Gamma point. The *d*-electrons of iron are treated as spin-polarized at all temperatures, densities, and compositions.

A pyrolite melt is used to model the composition of the bulk silicate Earth^16^. Minor elements are then added to the melt through substitution for Al^3+^ or Mg^2+^ while S is added either as S^2−^ through substitution for oxygen or as S^6+^ through the addition of an anhydrite component (see Supplementary Tables 1 and 2 for all melt compositions). To determine if S may affect the vaporization behavior of In, we add S to each In-bearing system in the form of sulfide (S^2−^) through atomic substitution for oxygen and we add S to the In^3+^-bearing system in the form of sulfate (S^6+^) through the addition of anhydrite. S was added in a ratio of 3:1 and 3:2 (S:In) to observe an effect on the vaporization behavior of In. However, we do not explore the effect of the concentration of S on the volatility of In. Future studies are needed to examine the effect of the In/S ratio and their absolute concentrations on the vaporization behavior of In.

We considered densities ranging from about 0.95 to 2.6 g cm^−3^. Nano-sized cavities begin to nucleate at ~2.6 g cm^−3^ and the total volume of the cavities increases with decreasing density. At a density of ~1.8 g cm^−3^, the more volatile elements, such as Na and In, begin to vaporize into the void space in the form of vapor molecules. This results in the coexistence of two phases: an interconnected silicate melt phase and a vapor-like phase. The speciation module of the Universal Molecular Dynamics package^41^ was used for identifying the vaporized species^42^. Maximum allowable bond distances were determined from the pair distribution functions^43^. The volatile species are able to continuously migrate between the void spaces and the silicate melt during the course of the simulation. The predisposition of the volatile species to exist in the vapor phase over the melt phase depends on the degree of volatility of the species and the pressure–temperature conditions. For one individual simulation, the volatilities are exact as they represent the number of atoms that exist in the vapor phase during the simulation time. Performing the simulations with different initial configurations and examining the convergence of the simulations with time gives an estimate of the uncertainties (Supplementary Table 4). Although a large fraction of the densities in this study corresponds to pressures below 10 kbar, ab initio molecular dynamics simulations are unable to accurately estimate low pressures due to the limitation in the size of the system (i.e., number of atoms) and the relatively short duration of the simulations^43,44^. Thus, we report the density–temperature conditions, which can be related to pressure through future experimental equation-of-state studies.

### Supplementary information

Supplementary Information

## Data Availability

All volatility data can be found at Zenodo (10.5281/zenodo.7133462). Parameters of the input files are described in Computational Methods.

## Code availability

Ab initio molecular dynamics simulations were performed with the Vienna Ab Initio Simulation Package (VASP), a proprietary software package available at https://www.vasp.at/. Post-processing of the resulting data was performed with the Universal Molecular Dynamics (UMD) package, distributed under GNU-GPL license and publicly available at https://github.com/rcaracas/UMD_package.
