# Supplementary material for: Earth’s volatile depletion trend is consistent with a high-energy Moon-forming impact
Source: Commun Earth Environ. 2023 Feb 16;4(1):38. doi: 10.1038/s43247-023-00694-9 (PMC11041689; doi:10.1038/s43247-023-00694-9)
Supplement: Supplementary file 1 — Supplementary Information [file 43247_2023_694_MOESM1_ESM.pdf]

Supplementary Information for

"Earth's Volatile Depletion Trend is Consistent with a High-Energy Moon-Forming Impact"

Natalia V. Solomatova*[1], Razvan Caracas[1,2,3]

[1] CNRS, Ecole Normale Supérieure de Lyon, Laboratoire de Géologie de Lyon LGLTPE UMR5276, Centre Blaise Pascal, 46 allée d'Italie Lyon 69364, France

[2] Université de Paris, Institut de Physique du Globe de Paris, CNRS, 1, rue Jussieu, Paris 75005, France

[3] The Center for Earth Evolution and Dynamics (CEED), University of Oslo, Blindern, Oslo, Norway

Supplementary Figures

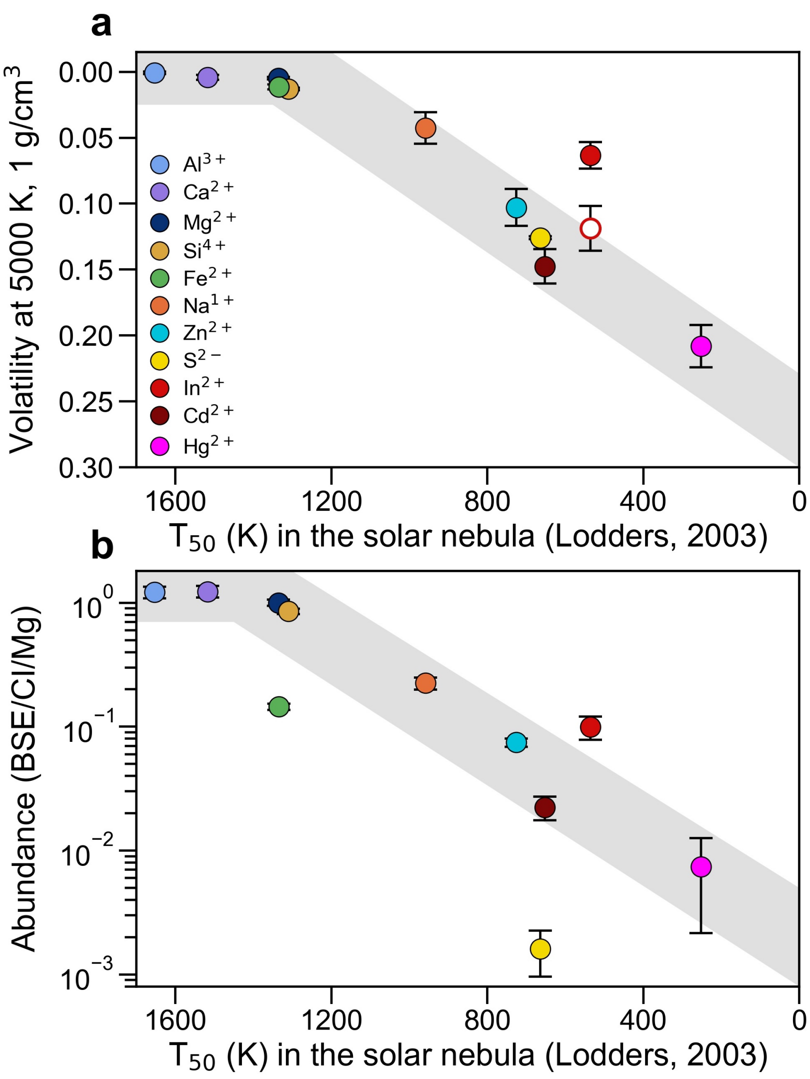

Supplementary Fig. 1 | Volatile depletion trends using the 50% condensation temperatures (T_{50}) in the solar nebula gas calculated by Lodders[1]. The overall trend and deviations are very similar to those in Fig. 2 where the reappraised 50% condensation temperatures of Wood et al.[2] were used. The calculated volatility of In^{2+} is shown in the absence of S^{2-} (red filled circle) and in the presence of S^{2-} (red open circle). Uncertainties on computed volatilities correspond to the convergence window of the volatilities with simulation time.

Supplementary Tables

Supplementary Table 1. Composition of the volatile-bearing pyrolite melts. Abundances are reported as oxide weight percentages. Here we report the compositions of all the volatile-bearing pyrolite melts considered in this study: (a) In^{1+}-bearing pyrolite melt, (b) In^{1+}-bearing pyrolite melt in the presence of S^{2-}, (c) In^{2+}-bearing pyrolite melt, (d) In^{2+}-bearing pyrolite melt in the presence of S^{2-}, (e) In^{3+}-bearing pyrolite melt, (f) In^{3+}-bearing pyrolite melt in the presence of S^{2-}, (g) In^{3+}-bearing pyrolite melt in the presence of S^{6+}, (h) Cd^{2+}-bearing pyrolite melt, (i) Zn^{2+}-bearing pyrolite melt, and (j) Hg^{2+}-bearing pyrolite melt. See Supplementary Table 2 for the stoichiometries.

	a	b	c	d	e	f	g	h	i	j
**SiO_2**	41.9	41.2	41.1	40.0	42.3	41.1	37.8	41.2	42.9	38.3
**MgO**	34.0	33.4	31.0	30.2	35.5	34.5	31.7	31.1	32.4	28.9
**CaO**	3.3	3.2	3.2	3.1	3.3	3.2	2.9	3.2	3.3	3.0
**Na_2O**	0.9	0.9	0.9	0.9	0.9	0.9	0.8	0.9	0.9	0.8
**Al_2O_3**	4.4	4.4	4.4	4.2	1.5	1.5	1.3	4.4	4.6	4.1
**FeO**	8.4	2.1	8.2	2.0	8.4	2.0	7.5	8.2	8.6	7.6
**FeS**	-	7.5	-	7.3	-	7.5	-	-	-	-
**InO**	-	-	11.2	-	-	-	-	-	-	-
**In_2O**	7.1	-	-	-	-	-	-	-	-	-
**In_2O_3**	-	-	-	-	8.1	-	7.3	-	-	-
**InS**	-	-	-	12.2	-	-	-	-	-	-
**In_2S**	-	7.5	-	-	-	-	-	-	-	-
**In_2S_3**	-	-	-	-	-	9.3	-	-	-	-
**$CaSO_4$**	-	-	-	-	-	-	10.7	-	-	-
**CdO**	-	-	-	-	-	-	-	11.0	-	-
**ZnO**	-	-	-	-	-	-	-	-	7.3	-
**HgO**	-	-	-	-	-	-	-	-	-	17.3

Note: The distribution of S^{2-} between InS and FeS (or any other species) is arbitrary, as the pyrolite melt is not physically synthesized from these oxides. We added a sufficient amount of S^{2-} to the system to observe its effect on the vaporization behavior of In.

Supplementary Table 2. Stoichiometry of the volatile-bearing pyrolite melts. Labels (a-j) correspond to the same labeling scheme as in Supplementary Table 1.

**a**	$NaCa_2Fe_4Mg_{29}Al_3In_2Si_{24}O_{89}$
**b**	$NaCa_2Fe_4Mg_{29}Al_3In_2Si_{24}S_4O_{85}$
**c**	$NaCa_2Fe_4Mg_{27}Al_3In_3Si_{24}O_{89}$
**d**	$NaCa_2Fe_4Mg_{29}Al_3In_3Si_{24}S_6O_{83}$
**e**	$NaCa_2Fe_4Mg_{30}AlIn_2Si_{24}O_{89}$
**f**	$NaCa_2Fe_4Mg_{30}Al_1In_2Si_{24}S_6O_{83}$
**g**	$NaCa_5Fe_4Mg_{30}Al_1In_2Si_{24}S_3O_{101}$
**h**	$NaCa_2Fe_4Mg_{27}Al_3Cd_3Si_{24}O_{89}$
**i**	$NaCa_2Fe_4Mg_{27}Al_3Zn_3Si_{24}O_{89}$
**j**	$NaCa_2Fe_4Mg_{27}Al_3Hg_3Si_{24}O_{89}$

Supplementary Table 3. A comparison of the 50% condensation temperatures. Here we report the 50% condensation temperatures, as calculated by Lodders[1] and Wood et al.[2] and the corresponding phases that were used for the calculations of the condensation temperatures. Forsterite, enstatite and clinopyroxene are abbreviated as fo, en, and cpx, respectively.

	Wood et al. (2019)		Lodders (2003)	
	T_{50} (K)	Phase	T_{50} (K)	Phase
Al	1652	corundum	1653	hibonite
Ca	1535	melilite	1517	hibonite + melilite
Mg	1343	fo + cpx	1336	fo
Si	1314	fo + cpx	1310	fo + en
Fe	1338	Fe alloy	1334	Fe alloy
Na	1035	plagioclase	958	feldspar
Zn	704	FeS	726	fo + n
S	672	FeS	664	FeS
In	492	FeS	536	FeS
Cd	502	FeS	652	en + FeS
Hg	240	FeS	252	FeS

Supplementary Table 4. Proportion of the element existing in the vapor phase at 5000 K and 1 g/cm^3. Oxidation states refer to the state in which the elements were added to the melt. The electronic structures are not constrained during the simulation and evolve over time as the local environment changes. Volatility values for In^{3+} and Cd^{2+} were extrapolated from the fitted volatilities, as shown in Figure 1 and error values were determined from the simulation at the closest density. Error values indicate the error on the percent volatility (e.g., the volatility of Na^{1+} is 4.2% +/- 0.9%) and correspond to the convergence window of the simulations.

Element	Volatility	Error	Element	Volatility	Error
Al^{3+}	0.1%	0.1%	In^{1+}	4.5%	1.2%
Ca^{2+}	0.4%	0.2%	In^{2+}	6.3%	1.0%
Mg^{2+}	0.5%	0.1%	In^{3+}	3.5%	0.6%
Si^{4+}	1.3%	0.1%	In^{1+} w/S^{2-}	6.6%	1.3%
Fe^{2+}	1.1%	0.2%	In^{2+} w/S^{2-}	11.9%	1.7%
Na^{1+}	4.2%	0.9%	In^{3+} w/S^{2-}	6.6%	0.4%
Zn^{2+}	10.3%	1.4%	In^{3+} w/S^{6+}	4.3%	0.5%
Cd^{2+}	14.8%	1.4%	S^{2-} w/In^{3+}	12.6%	0.2%
Hg^{2+}	20.8%	1.6%	S^{6+} w/In^{3+}	29.2%	1.6%

Supplementary Table 5. Types of vaporized species containing each volatile element at ~1 g/cm^3 and 5000 K. For the In^{3+}-bearing system, abundances are averaged over densities of 0.97 and 1.06 g/cm^3. Due to the low abundance of Al and Ca in the pyrolite system and their refractory nature (remaining almost entirely in the melt phase at 5000 K), their speciation in the vapor is not possible to accurately evaluate.

Mg^{2+}	Mg (35%), MgO (17%), $MgSiO_2$ (15%), $MgSiO_3$ (6%), $MgSi_2O_5$ (3%), MgO_2 (2%), $MgSiO_4$ (2%), $MgSi_3O_5$ (2%), $MgInSi_2O_6$ (2%), Mg_2FeSiO_6 (2%), $Mg_2Si_2O_6$ (2%)
Si^{4+}	SiO (42%), SiO_2 (26%), $MgSiO_2$ (9%), SiO_3 (4%), $MgSiO_3$ (3%), $CaSiO_2$ (2%), $MgSi_2O_5$ (2%), $MgSiO_4$ (1%), $MgSi_3O_5$ (1%), $MgSi_2InO_6$ (1%), $FeMg_2SiO_6$ (1%), $Mg_2Si_2O_6$ (1%), $Mg_2Si_3O_5$ (1%)
Fe^{2+}	FeO (34%), FeO_2 (22%), FeO_3 (17%), $FeMgO_3$ (5%), $FeMg_2SiO_6$ (5%), $FeMgO_2$ (4%), $FeSiO_2$ (3%), $FeSiO_4$ (3%), $FeMgSi_2O_6$ (2%), $FeMgSiO_5$ (2%), Fe (1%), $FeMg_2AlO_3$ (1%)
Na^{1+}	Na (76%), NaO_2 (18%), $NaSiO_3$ (1%), NaO (1%)
Zn^{2+}	Zn (93%), $SiZnO_2$ (2%), $SiZnO_3$ (2%), ZnO_2 (2%), ZnO (1%)
Cd^{2+}	Cd (90%), CdO (4%), CdO_2 (1%), $SiCdO_2$ (1%)
Hg^{2+}	Hg (87%), $SiHgO_2$ (3%), HgO (3%), $FeSiHgO_3$ (2%), HgO_2 (1%)
In^{1+}	In (76%), $InSiO_2$ (9%), InO (7%), $InSiO_3$ (4%), $InSi_2O_4$ (2%), $InMgSi_2O_5$ (2%)
In^{2+}	In (79%), InO (6%), $InSiO_2$ (5%), InSiO (3%), InO_2 (2%), $InMgSiO_4$ (2%), $InFeO_2$ (2%)
In^{3+}	In (69%), InO_2 (13%), InO (8%), InO_3 (4%), $InMgSi_2O_6$ (4%), InSiO (1%)
In^{1+} w/S^{2-}	In (84%), $InSiO_2$ (6%), InO (3%), InSiO (1%), InO_2S (1%), $InMgSiO_2$ (1%), InOS (1%)
In^{2+} w/S^{2-}	In (59%), In_2O (7%), InO_2S (7%), $InFeSiO_3$ (6%) InSiO (5%), InO (4%), $InMgSiO_4$ (3%), InO_3S (2%), $InAlSiO_3$ (2%) $InFeSi_2O_4$ (2%), InSiS (2%)
In^{3+} w/S^{2-}	In (59%), InO (7%), $InSiO_2$ (7%), InSiO (5%), InOS (4%), InO_2S (3%), FeInO (3%)
In^{3+} w/S^{6+}	In (77%), InO (8%), $MgInO_3$ (3%), $MgInO_2$ (2%), $InSiO_3$ (2%) and $InSiO_4$ (2%)
S^{2-} w/ In^{3+}	SO_2 (41%), SO (34%), S (7%), S_2 (2%), InOS (1%), MgSiOS (1%), MgO_2S (1%), MgOS (1%), InO_2S (1%)
S^{6+} w/ In^{3+}	SO_2 (39%), SO (29%), S (28%), SO_3 (1%)

Supplementary Notes

Supplementary Note 1

Indium exists in a range of oxidation states. With the presence of the highly reduced solar nebula gas, In likely existed in the +2 oxidation state[2]. The 50% condensation temperature for In has thus only been calculated for In^{2+} into InS, as it was likely the dominant In-bearing phase in the solar nebula gas during Earth's accretion[1,2]. However, In^{2+} is likely not a representative oxidation state after the Moon-forming impact, as the nebular gas would have dissipated within the first 3 million years[3] of the global magma ocean that existed after the Moon-forming impact was established by silicate-gas equilibria under much more oxidizing conditions compared to the solar nebula gas. In the present-day Earth, In exists almost exclusively in the +3 oxidation state, as seen in sulfide and oxide minerals, such as sphalerite[4,5], cassiterite[6,7], chalcopyrite[4,8] and stannite[6]. Furthermore, mineral-melt partitioning experiments have shown that In is incorporated into biotite and amphibole in the +3 oxidation state[9], strongly suggesting that In would have existed as In^{3+} in the early magma ocean. There have also been some studies exploring the +1 oxidation state[2,10,11]. In the melt-silicate partitioning experiments of Wang et al.[12], In was added as In_2O (+1 oxidation state) while in dissolution experiments on molten $CaO\text{-}SiO_2\text{-}Al_2O_3$ slags at highly reducing conditions, In was found to exist with the -1 and +1 oxidation states for silica contents of <20 wt% and >20 wt%, respectively[10,11]. However, it is unclear under what geological conditions In would exist in the -1 and +1 oxidation states.

Here, we compute the vaporization behavior of In added to pyrolite melt in the +3 oxidation state through substitution for Al^{3+} at densities of ~0.95 to 2.6 g/cm^3 and temperatures of 4000 K, 5000 K and 6000 K. To compare our results to previous studies, we also calculate the vaporization behavior of In added to pyrolite melt in the +1 and +2 oxidation states at 1 g/cm^3

and 5000 K. Our results indicate that In^{2+} is more volatile than In^{3+}, which may explain the apparent overabundance of In in the Earth relative to CI chondrites (see main text).

S is also found in various oxidation states, ranging from -2 and +6. Generally, S exists in the -2 oxidation state at log fO_2 < FMQ-1, in the +6 oxidation state at log fO_2 < FMQ+2 and in a multiple oxidation states at intermediate oxygen fugacity conditions[13-15]. The 50% condensation temperature for S has been calculated for the -2 oxidation state into FeS due to the reducing nature of the solar nebula gas. In this study, we consider the endmember oxidation states, S^{2-} and S^{6+}. We examine the effect of S^{2-} on the vaporization behavior of In^{1+}, In^{2+} and In^{3+} and the effect of S^{6+} on the vaporization behavior of In^{3+} at 5000 K and 1 g/cm^3. Although In and S are added to the system with a set oxidation state (e.g., In_2O, InO or In_2O_3), the electronic structures are not constrained throughout the simulation and evolve with time, depending on the local chemical environment.

Supplementary Note 2

The base pyrolite melt in our study is a six-component melt with a molar composition of $0.5Na_2O – 2CaO – 1.5Al_2O_3 – 4FeO – 30MgO – 24SiO_2$ where the six components are within 1 wt% of the nominal bulk silicate Earth composition[16]. The elemental concentration of In and Cd in the bulk silicate Earth are estimated as 11 and 40 ppb, respectively[16]. In this study we examine the partitioning behavior of trace elements between pyrolite melt and vapor. Due to computational feasibility, the pyrolite melts in our study contain 7 wt% In^{3+} and 10 wt% Cd^{2+} (melts “e” and “h” in Supplementary Tables 1 and 2), respectively. We have previously explored the effect of concentration on the partitioning behavior of volatile elements in pyrolite melt and did not observe a difference in vaporization behavior for the range of concentrations explored[17].

Ab initio molecular dynamics simulations are typically limited to 100-200 atoms due to the cubic-scaling cost associated with methods based on density functional theory. In these types of simulations, it is important to acquire sufficient statistics (i.e., to achieve convergence over time) in order to report meaningful vaporization proportions. Increasing the occurrence of the event of interest (i.e., the vaporization of the element) increases the statistical quality and decreases the error associated with the calculated vaporization proportion. This can be accomplished via longer simulation times and/or a larger concentration of the element of interest. Our low-density simulations (at 1 g/cm^3) with ~150-170 atoms produce about 200 time steps per day, parallelized on high-performance supercomputers. To achieve 20 ps, it thus takes 100 days of uninterrupted simulation time. To achieve 20 ps with a trace-element concentration that is even 1/2 or 1/4 of the present concentration, we would increase the number of atoms in our simulation 2-fold and 4-fold, which would result in a 8-fold and 64-fold increase in the required computational time, respectively.

With future advancements in machine-learning potentials based on *ab initio* molecular dynamics, it may become feasible to compute the vaporization behavior of trace elements in more realistic concentrations. Until then, the present study is a rigorous method based on first principles to calculate the melt-vapor partitioning behavior of elements with the available computational resources and technologies. The calculations performed in this study are novel and important for our understanding of the behavior of trace elements in silicate melts, and the results agree remarkably well with the experiments of Norris and Wood[18] on the volatility of In and Zn, providing a novel avenue for study of trace element volatility at Early Earth conditions.

Supplementary References

1. Lodders, K. Solar system abundances and condensation temperatures of the elements. *Astrophys. J.* **591**, 1220 (2003).
2. Wood, B. J., Smythe, D. J. & Harrison, T. The condensation temperatures of the elements: A reappraisal. *Am. Mineral.* **104**, 844-856 (2019).
3. Evans, N. J., Dunham, M.M., Jørgensen, J. K., Enoch, M. L., Merín, B., Van Dishoeck, E. F., Alcalá, J. M., Myers, P. C., Stapelfeldt, K. R., Huard, T. L. Allen, L. E., Harvey, P. M., Van Kempen, T., Blake, G. A., Koerner, D. W., Mundy, L. G., Padgett, D. L. & Sargent, A. I. The Spitzer c2d legacy results: star-formation rates and efficiencies; evolution and lifetimes. *Astrophys. J. Suppl. Ser.* **181**, 321 (2009).
4. Cook, N. J., Ciobanu, C. L. & Williams, T. The mineralogy and mineral chemistry of indium in sulphide deposits and implications for mineral processing. *Hydrometallurgy* **108**, 226-228 (2011).
5. Bauer, M. E., Seifert, T., Burisch, M., Krause, J., Richter, N. & Gutzmer, J. Indium-bearing sulfides from the Hämmerlein skarn deposit, Erzgebirge, Germany: evidence for late-stage diffusion of indium into sphalerite. *Miner. Depos.* **54**, 175-192 (2019).
6. Pavlova, G. G., Palessky, S. V., Borisenko, A. S., Vladimirov, A. G., Seifert, T. & Phan, L. A. Indium in cassiterite and ores of tin deposits. *Ore Geol. Rev.* **66**, 99-113 (2015).
7. Lerouge, C., Gloaguen, E., Wille, G., & Bailly, L. Distribution of In and other rare metals in cassiterite and associated minerals in Sn±W ore deposits of the western Variscan Belt. *Eur. J. Mineral.* **29**, 739-753 (2017).
8. George, L. L., Cook, N. J. & Ciobanu, C. L. Partitioning of trace elements in co-crystallized sphalerite–galena–chalcopyrite hydrothermal ores. *Ore Geol. Rev.* **77**, 97-116 (2016).

9. Gion, A. M., Piccoli, P. M. & Candela, P. A., Partitioning of indium between ferromagnesian minerals and a silicate melt. *Chem. Geol.* **500**, 30-45 (2018).

10. Ko, K. Y. & Park, J. H. Dissolution behavior of indium in $CaO-SiO_2-Al_2O_3$ slag. *Metall. Mater. Trans. B* **42**, 1224-1230 (2011).

11. Ko, K. Y. & Park, J. H. Dissolution mechanism of indium in $CaO-Al_2O_3-SiO_2$ slag at low silica region. *Metall. Mater. Trans. B* **43**, 440-442 (2012).

12. Wang, Z., Laurenz, V., Petitgirard, S. & Becker, H. Earth's moderately volatile element composition may not be chondritic: Evidence from In, Cd and Zn. *Earth Planet. Sci. Lett.* **435**, 136-146 (2016).

13. Jugo, P. J., Wilke, M. & Botcharnikov, R. E. Sulfur K-edge XANES analysis of natural and synthetic basaltic glasses: Implications for S speciation and S content as function of oxygen fugacity. *Geochim. Cosmochim. Acta* **74**, 5926-5938 (2010).

14. Nash, W. M., Smythe, D. J. & Wood, B. J. Compositional and temperature effects on sulfur speciation and solubility in silicate melts. *Earth Planet. Sci. Lett.* **507**, 187-198 (2019).

15. Wang, W., Li, C. H., Brodholt, J. P., Huang, S., Walter, M. J., Li, M., Wu, Z., Huang, F. & Wang, S. J. Sulfur isotopic signature of Earth established by planetesimal volatile evaporation. *Nat. Geosci.* **14**, 806-811 (2021).

16. Behrens, H., & Gaillard, F. Geochemical aspects of melts: volatiles and redox behavior. *Elements* **2**, 275-280 (2006).

17. Solomatova, N. V. & Caracas, R. Genesis of a CO_2-rich and H_2O-depleted atmosphere from Earth's early global magma ocean. *Sci. Adv.* **7**, eabj0406 (2021).

18. Norris, C. A. & Wood, B. J. Earth's volatile contents established by melting and vaporization. *Nature* **549**, 507-510 (2017).
